# Enhancement of Mechanical Properties of Zein-Based Nanofibers by Incorporation of Millet Gliadin

**DOI:** 10.3390/foods13182900

**Published:** 2024-09-13

**Authors:** Shumin Wang, Pengjie Wang, Siyuan Liu, Ran Wang, Yixuan Li, Xiaoyu Wang, Fazheng Ren, Jie Luo, Bing Fang

**Affiliations:** 1 Key Laboratory of Precision Nutrition and Food Quality, Department of Nutrition and Health, China Agricultural University, Beijing 100083, China; sdutshumin@163.com (S.W.); wpj1019@cau.edu.cn (P.W.); siyuan.liu@cau.edu.cn (S.L.); wangran@cau.edu.cn (R.W.); liyixuan@cau.edu.cn (Y.L.); xy.wang@cau.edu.cn (X.W.); renfazheng@cau.edu.cn (F.R.); 2 Department of Food and Bioengineering, Beijing Vocational College of Agriculture, Beijing 102442, China; 3 Food Laboratory of Zhongyuan, Luohe 462300, China; 4 College of Food Science and Technology, Hunan Agricultural University, Changsha 410114, China

**Keywords:** electrospinning, millet gliadin, zein, mechanical properties

## Abstract

In this work, a novel reinforcing filler, millet gliadin (MG), was used for the improvement of the mechanical properties of zein nanofibers. The structural and physicochemical properties of MG were compared with those of zein, and the influence of MG on the morphology, physical properties, and molecular structure of zein nanofibers was investigated. The results indicated that MG has an obviously smaller weight-average molecular weight (7623) in comparison to zein (13,330). Transmission electron microscopy showed that zein molecules more easily form aggregates with larger diameters than MG molecules in acetic acid. At a concentration of 30% (*w*/*v*), MG exhibited a significantly higher viscosity (0.66 ± 0.03 Pa·s) than zein (0.32 ± 0.01 Pa·s), indicating the stronger interactions of MG molecules. With the incorporation of MG, the tensile strength was significantly increased to 49.32 MPa (ZM-1/2), which is 2.08 times and 4.45 times higher than that of pure zein nanofibers (ZM-1/0) and MG nanofibers (ZM-0/1-1), respectively. Moreover, zein/MG composite nanofibers exhibited improved water stability. Fourier transform infrared spectra showed evidence of the hydrogen bonding interaction between zein and MG. Therefore, MG is a good candidate for use as a natural reinforcing filler in electrospun nanofibers made of biopolymers.

## 1. Introduction

Electrospinning is a simple, low-cost, and effective technology for producing nonwoven fibers that possess high pore interconnectivity and large surface area-to-volume ratios. Therefore, electrospun nanofibers have potential in many applications such as food packaging [1], filtration [2], wound healing [3], metal ion removal [4], and bioactive delivery [5]. Natural biopolymers are considered excellent raw materials for electrospun nanofibers because of their renewability, biodegradability, and biocompatibility. As fundamental building blocks of life, proteins are crucial for the elasticity, stability, and protection of cells, tissues, and organisms [6]. Therefore, electrospun nanofibers made of proteins are considered excellent candidates for utilization in various fields such as biomedicine, food packaging, and cosmetics. However, protein nanofibers are generally constrained by their poor mechanical properties [7]. Therefore, it is necessary to reinforce electrospun protein nanofibers.

According to the existing literature, cross-linking or fabricating assembled nanofibers is reported to enhance the mechanical properties of protein nanofibers. Physical cross-linking [8] and chemical cross-linking by agents such as xylose [9], hexamethylene diisocyanate [10], genipin [11], and glutaraldehyde [12] have been performed to reinforce protein nanofibers. However, the existing cross-linking approaches are either ineffective, cytotoxic, or expensive [12,13]. On the other hand, incorporating polymers [14,15] or natural biopolymers into the matrix is considered to overcome the limitation in mechanical strength. However, the polymers have been found to release toxic substances during degradation, which may lead to inflammatory reactions [16]. Therefore, biopolymers that are naturally extracted are highly appealing for the reinforcement of protein nanofibers. The mechanical properties of hordein/zein nanofibers are reported to be successively improved by the incorporation of cellulose nanowhiskers [6]. In a study by Wang et al. [7], zein was used as the reinforcing filler in hordein fibers, which exhibited improved tensile strength in contrast to the neat hordein nanofibers. But studies on the application of biopolymers to strengthen protein-based nanofibers are still very limited. Thus, the development of novel natural polymers as reinforcement fillers is of great importance for fabricating protein nanofibers with good performance.

Zein is known to be a promising material for electrospinning; the obtained nanofibers have been demonstrated to be utilizable as food packaging layers, encapsulant of active substances, and excipient of pharmaceuticals [17,18,19]. However, the weak mechanical strength of zein nanofibers is still a concern in their application [15]. Millet gliadin, which is prolamin originating from millet grains, accounts for about 60% of the millet’s protein [20]. Furthermore, millet gliadin is rich in nutrients and has shown anti-diabetic effects [21]. Current research into millet gliadin is very limited, mainly focusing on its extraction, physicochemical property characterization, and beneficial effect evaluation [21,22,23,24,25], but efforts towards the fabrication of electrospun nanofibers and their use in reinforcing protein nanofibers are lacking. Similar to zein, millet gliadin has good hydrophobicity as well as film-forming properties, which contribute to their excellent compatibility and potential interactions. Moreover, millet gliadin has been found to have good digestive stability and thermal stability [26]. Prolamins are said to be excellent materials for producing nanofibers with good mechanical properties [7,16]. As one of the main members of the prolamin family, millet gliadin may be a promising material for improving the mechanical strength of electrospun nanofibers. Therefore, we hypothesize that blending zein with millet gliadin is an effective strategy for improving the mechanical strength of electrospun zein nanofibers.

To confirm this hypothesis, in this work, millet gliadin was extracted and applied as reinforcement in zein matrices to fabricate bicomponent nanofibers. The structural and physicochemical properties of zein and millet gliadin were characterized. The effects of using millet gliadin as reinforcement on the morphology, mechanical properties, water resistance, and molecular structure of zein nanofibers were then investigated. Moreover, the formation mechanism of electrospun zein/millet gliadin nanofibers was explored.

## 2. Materials and Methods

### 2.1. Materials

Zein (92 wt% protein content and ~20 kDa molecular weight) was purchased from Sigma-Aldrich (St. Louis, MO, USA). Millet grains (Jinmiao K1, 9.6 wt% protein content) were obtained from JD supermarket. Millet gliadin (90.8 wt% protein content and 3.27 wt% yield) was extracted using an ethanol protocol. Glacial acetic acid, ethanol, methanol, hexane, and tetrahydrofuran were purchased from Sinopharm Chemical Reagent Co., Ltd. (Beijing, China).

### 2.2. Millet Gliadin Isolation from Millet Grains

Zein/millet gliadin was prepared according to previous research, with some modifications [9,27]. Millet grains were ground to millet flour using laboratory milling equipment. The millet flour was defatted with hexane at a millet flour-to-hexane ratio of 1:5 (*w*/*v*), and the defatted millet flour was obtained after centrifugation at 8000× *g* at 25 °C for 10 min. The defatted millet flour was then blended with distilled water at a ratio of 1:10 (g/mL). The mixture was mixed thoroughly and processed in a colloid mill for 15 min. After the suspension was mixed using a stirrer for 30 min at 25 °C, it was centrifuged at 3500× *g* for 10 min. The supernatant was removed, and ethanol (80%, *v*/*v*) was added at a ratio of 1:8 (g/mL). After the mixture was stirred at 37 °C for 3 h, it was centrifuged for 10 min at 3500× *g*. Sodium chloride solution was slowly added to the obtained supernatant while stirring until the volume was three times that of the original supernatant and the final concentration of sodium chloride reached 0.3% (*w*/*v*). The mixture was allowed to stand at 4 °C for 24 h to cause the millet gliadin to precipitate out. Finally, millet gliadin was obtained after centrifugation, water washing, and freeze-drying.

### 2.3. Characterization of Zein and Millet Gliadin

#### 2.3.1. Sodium Dodecyl Sulfate Polyacrylamide Gel Electrophoresis (SDS-PAGE)

SDS-PAGE was conducted through a modified method according to Zhang et al. [28]. Protein solutions (2 mg/mL) in distilled water (pH 12) were mixed with 5× loading buffer in a boiling water bath for 5 min. After cooling, SDS-PAGE was carried out by loading 10 μL of each sample into gels consisting of a 5% stacking gel and a 12% separating gel. The voltage of the stacking gel was set to 120 V, and the voltage of the separating gel was set to 80 V. The electrophoresis ended when bromophenol blue was about 1 cm from the bottom of the gel. Finally, the gels were dyed with Coomassie blue R-250 for 30 min and de-stained overnight in a 7.5% methanol and 5% acetic acid solution.

#### 2.3.2. Gel Permeation Chromatography (GPC)

The molecular weights of zein and millet gliadin were measured by GPC according to the protocol reported by Zhang et al. [28], with some modifications. A gel column of Shodex KD-806M was used. The mobile phase used tetrahydrofuran containing 0.064 mol/L NaNO_3_. The molecular weights of the zein and millet gliadin were determined by isocratic elution under the chromatographic conditions of 1 mL/min flow rate, 220 nm detection wavelength, and 30 °C column temperature.

#### 2.3.3. Amino Acid Composition

The amino acid compositions of the zein and millet gliadin were examined as reported by Balaguer, Gomez-Estaca, Gavara, and Hernandez-Munoz [29]. The zein and millet gliadin powders were separately dissolved in 6 mol/L hydrochloric acid solution containing 0.1% phenol. The samples (3 mL) were hydrolyzed in vacuum-sealed glass tubes at 110 °C for 24 h. They were then vacuum-dried and dissolved in distilled water. The amino acid composition was studied by high-performance liquid chromatography with post-column derivatization.

#### 2.3.4. Micromorphology of Zein and Millet Gliadin

The appropriate amount of zein or millet gliadin powder sample was fixed to the surface of the platform using a double-sided guide tape, and the resident samples were removed. Then, the samples were sputter-coated with gold for 60 s under vacuum. The micromorphology of the zein and millet gliadin powder samples was observed with an SU8020 scanning electron microscope (Hitachi, Tokyo, Japan) at 1000× and 2000× magnification.

The morphology of zein and millet gliadin in acetic acid at a concentration of 0.1 mg/mL was observed using a JEM-1200EX transmission electron microscope (JEOL, Tokyo, Japan). A drop of the protein solution was deposited on a copper grid. The specimen was then dried in air at ambient temperature (25 ± 1 °C).

#### 2.3.5. Intrinsic Fluorescence Spectroscopy

The intrinsic fluorescence spectra of the zein (0.2 mg/mL in 80% ethanol, *v*/*v*) and millet gliadin samples (0.2 mg/mL in 80% ethanol, *v*/*v*) were measured with an RF-5301PC fluorescence spectrophotometer (Shimadzu, Kyoto, Japan) at 25 °C. An excitation wavelength of 280 nm, emission wavelength range of 250–450 nm, slit width of 5 nm, and scanning speed of 120 nm/min were used.

#### 2.3.6. Fourier Transform Infrared (FTIR) Spectroscopy

Infrared spectra of the zein and millet gliadin powder samples were recorded on a Nicolet 6700 FTIR spectrometer (Thermo Scientific, Waltham, MA, USA) at 25 °C. The powder samples were mixed with potassium bromide (KBr) at a ratio of 1:100 (*g*/*g*), ground using an agate mortar into a fine powder, and then pressed into tablets for FTIR spectrum scanning. The wavenumber range was 4000–400 cm^−1^, with a resolution of 4 cm^−1^. Each measurement used an average of 32 scans. The secondary structures (α-helix, β-sheet, β-turn, and random coil) of the zein and millet gliadin samples were determined on the basis of the amide I band (1700–1600 cm^−1^). Curve-fitting analysis was conducted using PeakFit v.4.12 software (Seasolve Software, Framingham, MA, USA). Gaussian deconvolution and second derivative were performed using OMNIC 9.2 software (Thermo Fisher Scientific Inc., Carlsbad, CA, USA).

#### 2.3.7. Viscosity of Zein and Millet Gliadin in Acetic Acid

The zein and millet gliadin powder samples (30%, *w*/*v*) were separately dissolved in acetic acid, and the dispersions were stirred overnight at 25 °C using a magnetic stirrer (MYP11-2, Shanghai, China). The viscosity test was performed using a AR2000ex Rotational rheometer (TA Instruments, New Castle, DE, USA). The viscosity of the solutions was measured with the steady state flow mode and by ramping the shear rate from 0.01 s^−1^ to 200 s^−1^. The viscosity of solutions was reported at 100 s^−1^. All measurements were carried out at ambient temperature (25 ± 2 °C).

### 2.4. Preparation of Electrospinning Nanofibers

The sample components are listed in Table 1. It should be noted that the formulations are designed based on similar fiber diameters (200–300 nm). Hence, the concentrations of protein in different formulations are not equal, except for ZM-1/0 and ZM-0/1-2. In our previous work, fiber diameters were found to greatly affect the release behavior, surface wettability, and water stability of electrospun zein nanofibers. To eliminate the possible influence of fiber diameter on the physicochemical properties (including mechanical properties) of nanofiber samples, the zein/millet gliadin composite nanofibers were fabricated under the premise of similar fiber diameters.

The zein and millet gliadin powder samples were dissolved in acetic acid, and the dispersions were stirred overnight at 25 °C using a magnetic stirrer. The protein solutions were then allowed to stand for 10 min to remove bubbles. A 10 mL syringe was filled with the protein solution, and nanofibers were produced using an electrospinning device (HZ-11, Qingdao, China). The processing parameters used were as follows: 20 kV electrical voltage, 0.5 mL/h flow rate, 1000 rpm target roll speed, and 10 cm tip-to-collector distance. The solution volume of each sample remained constant at 2 mL. The nanofiber samples with different millet gliadin concentrations were labeled ZM-1/0, ZM-2/1, ZM-1/1, ZM-1/2, ZM-0/1-1, and ZM-0/1-1, respectively.

### 2.5. Scanning Electron Microscopy (SEM)

Morphology observations of the electrospun nanofibers were performed with a scanning electron microscope (Hitachi, Tokyo, Japan) at an acceleration voltage of 10 kV after sputter coating with gold. ImageJ v1.8.0 software was used to investigate the fiber diameters. Two hundred random fibers were selected and determined for each sample. The fiber diameter distribution and span were obtained according to the following formulas [30]:

Fiber diameter distribution = (Fiber diameter standard deviation/Fiber diameter)^2^, (1)

Span = (D_v90_ − D_v10_)/D_v50_. (2)

In Equation (2), D_v90_, D_v50_, and D_v10_ indicate the point in the size distribution, up to and including 90%, 50%, and 10%, respectively, of the fiber diameters [30].

### 2.6. Tensile Testing

The nanofibers (thickness of 0.02 mm) were cut into 1 cm × 3 mm (length × width) pieces, and then tensile testing was carried out using a universal testing machine (Instron, Norwood, MA, USA) [31]. The crosshead speed and gauge length were 5 mm/min and 5 mm, respectively. Five assays of each nanofiber sample were conducted. The tensile strength (TS), elongation at break (EB), and Young’s modulus (YM) were obtained according to the following formulas:

TS = F_m_/S, (3)

EB = ((L_b_ − L_0_)/L_0_) × 100%, (4)

YM = (F_m_ × L_g_)/(S × L_m_), (5)

where F_m_ is the maximum force (N) recorded, S is the cross-sectional area of the nanofibers, L_b_ is the length (mm) at breaking point, L_0_ is the initial length of the nanofibers, L_m_ is the test length (mm) corresponding to the maximum force, and L_g_ is the gauge length (mm).

### 2.7. Water Contact Angle (WCA)

The WCAs of the electrospun nanofibers on the glass slide were determined using an OCA 25 contact angle meter (DataPhysics Instruments GmbH, Filderstadt, Germany) according to the method reported by Wang et al. [31]. Three microliters of distilled water was dripped onto each nanofiber, and then the WCA was recorded within 5 s by the meter. Determinations were replicated five times for each nanofiber sample.

### 2.8. Water Stability

The water stability of the zein and millet gliadin nanofibers was evaluated according to the method reported by Lu, Wang, Li, Qiu, and Wei [32]. Samples of the zein and millet gliadin nanofibers were cut into 2.5 cm × 2.5 cm pieces and then immersed in 35 mL deionized water in petri dishes (90 mm diameter) for 24 h. Images of the test samples before and after immersion in water were taken to demonstrate the changes in the nanofibers. In addition, the soaked nanofibers were freeze-dried, and the morphological changes were observed by SEM.

### 2.9. Thermal Property

Thermogravimetric analysis was performed using an STA 449 F5 instrument (Netzsch-Gerätebau GmbH, Selb, Germany) according to previous research [33]. The weight loss of nanofiber samples in the temperature range of 25–600 °C was measured at a heating rate of 10 °C/min, and the whole process was carried out under a nitrogen atmosphere (50 mL/min).

### 2.10. Attenuated Total Reflectance Infrared Spectroscopy (ATR-FTIR)

The functional groups and secondary structures of the nanofiber samples were investigated with a Nicolet 6700 FTIR spectrometer (Thermo Scientific, Waltham, MA, USA) with an ATR unit attached. Infrared spectra were recorded in the wavenumber range of 4000–400 cm^−1^ and at a resolution of 4 cm^−1^, with an average of 32 scans. The amide I band (1700–1600 cm^−1^) of the spectra was used to evaluate the secondary structure of the nanofiber samples by using PeakFit v.4.12 software (Seasolve Software, Framingham, MA, USA) and OMNIC 9.2 software (Thermo Fisher Scientific Inc., Carlsbad, CA, USA).

### 2.11. Statistical Analysis

All determinations were made at least in triplicate, and the results were presented as mean ± standard deviation. Statistical analysis was conducted using single-factor analysis of variance and Duncan’s multiple range test, using SPSS 20 software and with a significance level of 0.05 (*p* < 0.05).

## 3. Results and Discussion

### 3.1. Composition and Structure of Zein and Millet Gliadin

#### 3.1.1. Molecular Weight Profile and Amino Acid Composition

**Figure 1:**
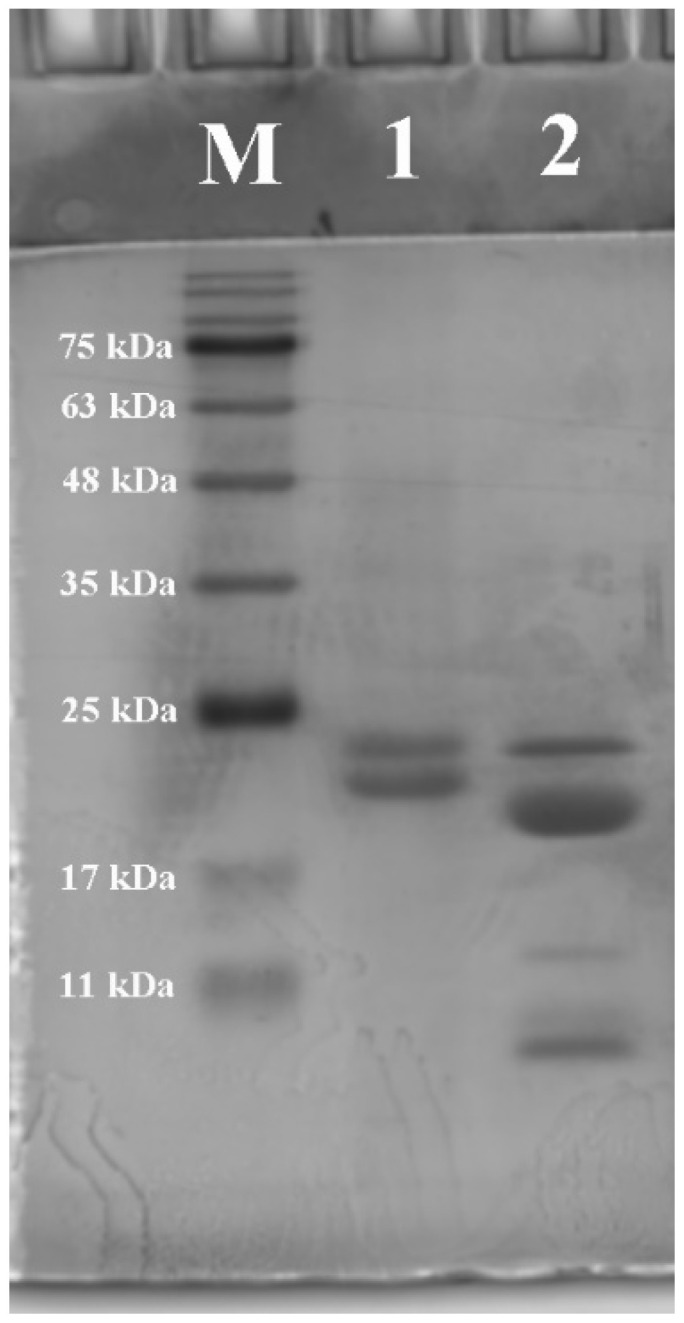
Sodium dodecyl sulphate polyacrylamide gel electrophoresis (SDS-PAGE) image of zein and millet gliadin. M: marker, lane 1: zein, lane 2: millet gliadin.

SDS-PAGE and GPC were performed to analyze the molecular weights of zein and millet gliadin. As shown in Figure 1, the main bands of zein (lane 1) have molecular weights of about 21 kDa and 23 kDa, suggesting that the commercially available zein used in this study is mainly composed of α-zein. Zhang et al. [28] also reported similar results. The millet gliadin mainly consists of four bands with molecular weights of 11, 13 (β-prolamin), 20 (α-prolamin), and 23 kDa (γ-prolamin), and one major band has a molecular weight of 20 kDa (lane 2), which has been proven to be homologous in all millets [21]. This finding is consistent with the results of the previous literature [20,34]. In addition, GPC was used to characterize the relative molecular weights of zein and millet gliadin. It can be seen from Table 1 that the weight-average molecular weight (M_w_) of millet gliadin is 7623, which is less than that of zein (13,330).

The alcohol-soluble proteins of cereal grains were given the name prolamin because of their high contents of proline and glutamine. To understand the variety of prolamin protein structures, amino acid analysis of zein and millet gliadin was performed. As described in Table 2, millet gliadin exhibits more balanced profiles for amino acids than zein does, although the glutamic acid content is much higher than that of the other amino acids. Furthermore, in comparison to zein, millet gliadin is richer in aspartic acid, methionine, and cysteine, while its leucine, glutamate, and proline levels are lower. According to previous reports, leucine is a non-polar amino acid with an aliphatic R group, and glutamic acid is generally involved in the formation of hydrogen bonds, while cysteine and methionine can contribute to the formation of disulfide bonds [35]. Therefore, it can be inferred that millet gliadin has more intramolecular disulfide bonds, weaker hydrophobic interactions, and fewer intermolecular hydrogen bonds than zein. Furthermore, compared with zein, millet gliadin exhibits fewer hydrophobic amino acids, indicating that millet gliadin has higher hydrophilicity.

#### 3.1.2. Structural Properties

**Figure 2:**
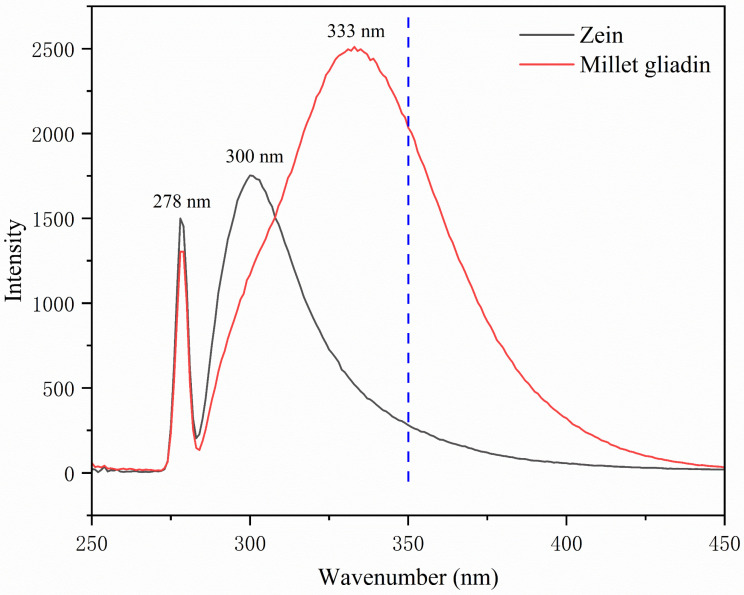
Endogenous fluorescence spectra of zein and millet gliadin.

Fluorescence spectra are regarded as a sensitive means of characterizing protein conformation and structural information. Aromatic residues, including tyrosine, tryptophan, and phenylalanine, that are present in proteins fluoresce in a way that depends on the folding of the protein. Therefore, fluorescence spectra can reflect the conformation in the tertiary structure of protein molecules [36,37]. Intrinsic fluorescence spectra of zein and millet gliadin are presented in Figure 2. Zein exhibits a fluorescence emission maximum of around 300 nm, which is mainly attributed to the higher content of tyrosine residues than tryptophan residues in zein [38]. Joye, Davidov-Pardo, Ludescher, and McClements [39] also observed similar results. The fluorescence intensity of millet gliadin is the strongest, at 333 nm, which is similar to the emission maximum (~336 nm) reported for gliadin [39], indicating that the tryptophan residues contribute the most among the three chromophoric amino acids. In general, the tryptophan residues are believed to be “buried” in a “non-polar” environment when the maximum emission wavelength (λ_max_) is less than 330 nm. On the contrary, the tryptophan residues are indicated to be in a “polar” environment [24,40]. Therefore, the tryptophan residues of millet gliadin are in a polar environment, while those of zein are in a non-polar environment. These results indicate the fact that millet gliadin molecules are more extended than zein molecules in solution, allowing more hydrophobic groups inside the molecules to be exposed, thus increasing the polarity of tryptophan residues. Moreover, compared with zein, millet gliadin shows a higher emission fluorescence intensity (FI), which might be attributed to the higher content of tryptophan in millet gliadin. In a previous study, the author reported that the λ_max_ of zein occurred at 348 nm, suggesting that the tryptophan residues of zein were in a polar environment [40]. This inconsistent result is probably due to different solvents being used.

Fluorescence FTIR was employed to evaluate the major conformation of the secondary structures of zein and millet gliadin (Figure S1). It has been reported that the C=O stretching vibration at 1700–1600 cm^−1^ (amide I band) is particularly sensitive to protein conformation; hence, the secondary structure content was measured through deconvolution of the amide I band in the FT-IR spectra. The corresponding relationship between peak area and secondary structure content is based on the following values: α-helix at 1659–1648 cm^−1^, β-sheet at 1700–1680 cm^−1^ and 1640–1610 cm^−1^, β-turn at 1679–1660 cm^−1^, and random coil at 1648–1640 cm^−1^ [41]. As shown in Table 3, the secondary structure composition of zein is 25.40% α-helix, 32.63% β-sheet, 18.56% β-turn, and 23.40% random coil. For millet gliadin, β-sheet and β-turn content is increased, while random coil content is decreased. The increase in β-sheet and β-turn content may be a sign of the loosening of the millet gliadin molecules [37]. The decrease in random coil content suggests that the secondary structure of millet gliadin is more ordered and stable than that of zein [24].

#### 3.1.3. Morphology

**Figure 3:**
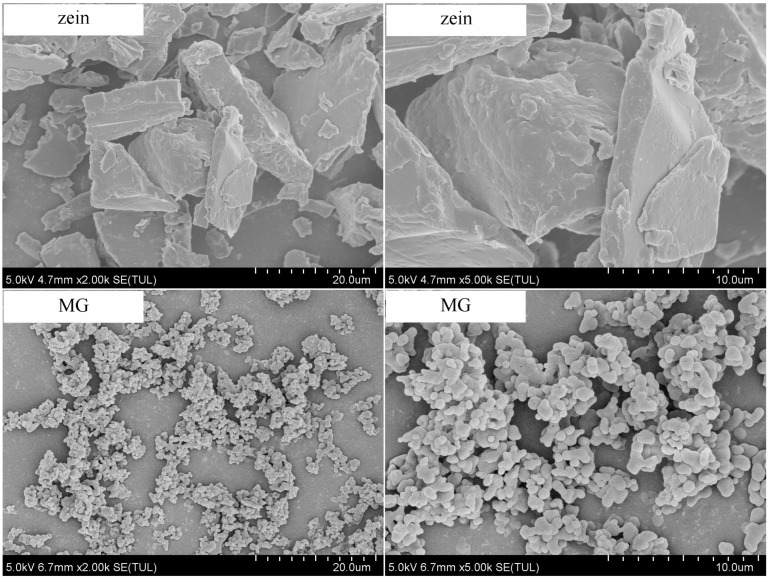
Scanning electron microscopy images of zein and millet gliadin.

Figure 3 shows the surface morphologies of zein and millet gliadin in the powder state. Millet gliadin presents a relatively regular spherical shape, while zein particles appear in irregular sizes and various shapes. In a study by Zhang et al. [24], spherical prolamins in foxtail millet were also observed.

**Figure 4:**
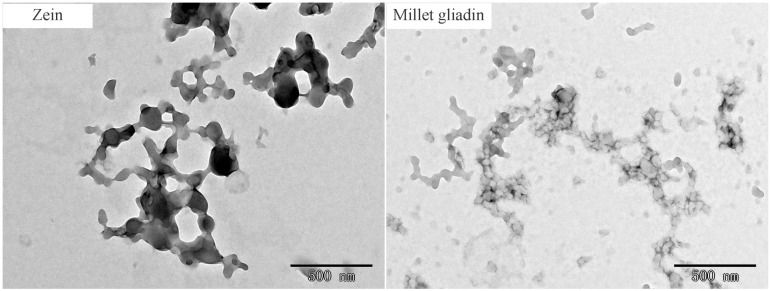
Transmission electron microscope images of zein and millet gliadin in acetic acid.

Acetic acid is an appropriate solvent for prolamin proteins in the production of electrospun fibers. To study the molecular conformations of millet gliadin and zein, the aggregation structures of these proteins in dilute solutions were observed using a transmission electron microscope. As seen in Figure 4, millet gliadin and zein present spherical and compact aggregates, but zein particles have a larger diameter. These results indicate that zein molecules aggregate more easily than millet gliadin molecules. The larger diameter of the zein particles might be attributed to their higher molecular weight, as well as the fact that there are more hydrogen bonds and hydrophobic interactions in the molecules of zein.

### 3.2. Solution Viscosity

The viscosity of both millet gliadin and zein in acetic acid was determined at 25 °C. At the same concentration, millet gliadin exhibited a significantly higher viscosity (0.66 ± 0.03 Pa·s) than zein (0.32 ± 0.01 Pa·s), indicating the more extended structure of millet gliadin and its strong interaction with acetic acid. As reported, acetic acid could fully unfold the β-sheet structure and partially disrupt the α-helix structure of protein molecules [24]. Compared with zein, millet gliadin exhibited a higher β-sheet content; therefore, the dissolved millet gliadin possessed a more extended and elongated structure in acetic acid, allowing millet gliadin molecules to form strong interactions more easily.

### 3.3. Nanofiber Characterization

#### 3.3.1. Morphology and Diameter Distribution of Nanofibers

**Figure 5:**
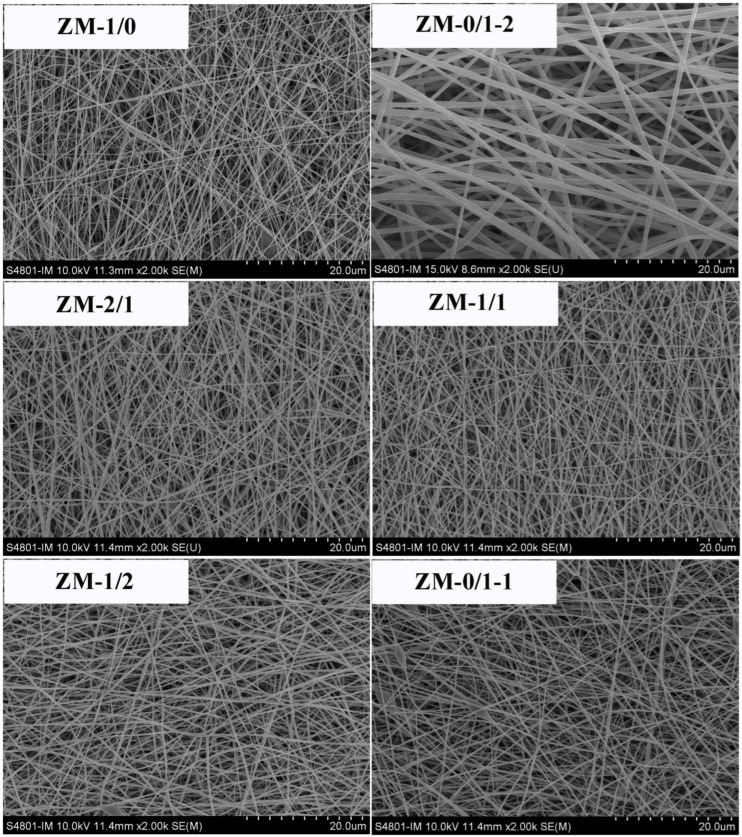
Scanning electron microscopy images of zein/millet gliadin composite nanofibers.

Figure 5 exhibits the SEM images of zein/millet gliadin composite nanofibers. The fiber diameter, diameter distribution, and span are displayed in Table 4. Fibers in all nanofiber samples were smooth, uniform, bead-free, and randomly oriented. Furthermore, there was no significant phase segregation, confirming excellent compatibility between zein and millet gliadin. According to our previous research, the optimized electrospinning concentration for zein is 300 mg/mL, and millet gliadin showed good electrospinnability at that concentration. However, compared with the zein nanofibers (ZM-1/0, 208.22 ± 47.04 nm, 0.64), the millet gliadin nanofibers (ZM-0/1-2) exhibited an obviously larger diameter (634.79 ± 152.73 nm) and span (0.73). These results are related to the higher viscosity of the millet gliadin solution, suggesting stronger intermolecular interactions of millet gliadin in solution. To eliminate the influence of fiber diameter, zein/millet gliadin composite nanofibers with diameters between 265 and 299 nm were prepared. Fiber diameter distribution is a determining factor of the homogeneity of nanofibers. The smallest fiber diameter distributions were observed in ZM-1/1 and ZM-1/2.

#### 3.3.2. Mechanical Properties

**Figure 6:**
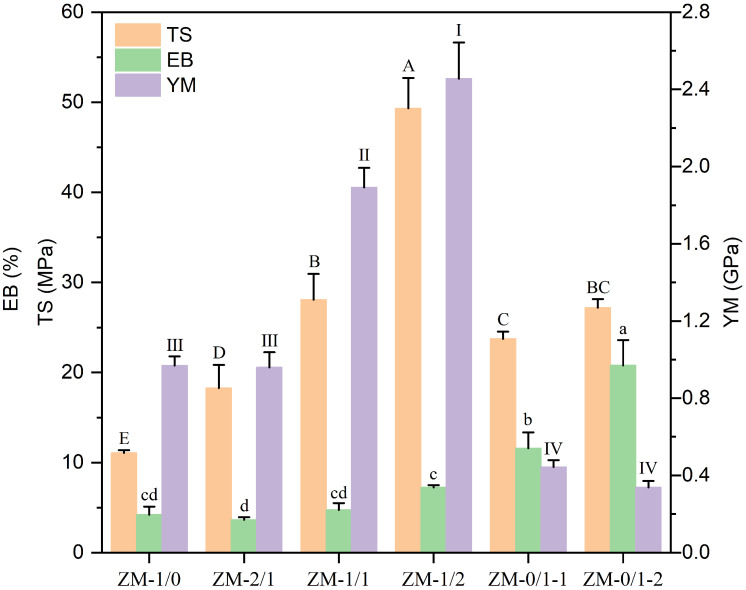
Mechanical properties of zein/millet gliadin nanofibers. Various symbols on top of columns suggest significant differences (*p* < 0.05).

As reported previously, the mechanical properties of nanofibers are closely related to their protein conformation [11]. To reveal the internal structures and to evaluate the handling properties, the mechanical properties of composite nanofibers with different zein/millet gliadin ratios were measured. As shown in Figure 6, the TS, EB, and YM values of the zein nanofiber (ZM-1/0) are 11.08 MPa, 4.21%, and 0.97 GPa, respectively. A higher TS (23.72, and 27.19 MPa) and EB (11.58, and 20.78%) and a lower YM (0.44, 0.34 GPa) are observable for the millet gliadin nanofibers (ZM-0/1-1, ZM-0/1-2), suggesting that millet gliadin nanofibers have significantly enhanced mechanical properties. The different results between ZM-0/1-1 and ZM-0/1-2 may be related to their distinct fiber diameters [42]. Upon the change in the zein/millet gliadin ratio from 1:0 to 1:1 and 1:2, the TS increases drastically from 11.08 MPa to 28.08 and 49.32 MPa, and the YM increases from 0.97 GPa to 1.89 and 2.45 GPa. By incorporating millet gliadin into the zein network, the fiber strength and stiffness are greatly improved. This might be attributed to the strong interaction between zein and millet gliadin at those zein/millet gliadin ratios, as that interaction could stabilize protein structures during the electrospinning process. Wang et al. [7] also observed that hordein/zein composite nanofibers exhibited significantly improved mechanical properties compared to pure hordein nanofibers. In the work of Wang et al. [38], an increased TS of zein nanofibers from 7.86 MPa to 18.27 MPa was observed after the addition of chlorogenic acid (2 wt%). Previously, Lu et al. [32] reported that composite nanofibers with a zein/ethyl cellulose ratio of 1:1 had a TS of 5.77 MPa, higher than that of pure zein nanofibers (5.12 MPa) or ethyl cellulose nanofibers (1.05 MPa).

#### 3.3.3. Surface Properties of Nanofibers

**Figure 7:**
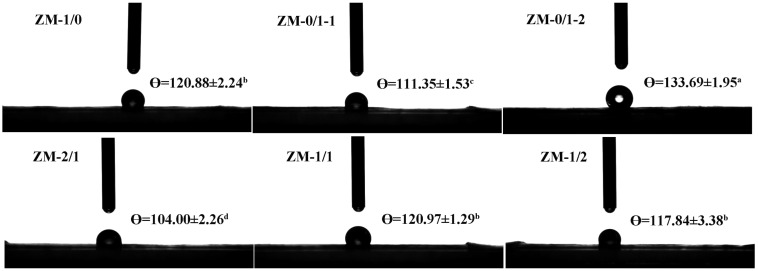
Images of water contact angle determinations for zein/millet gliadin nanofibers. Results with different lowercase superscript indicate significant differences (*p* < 0.05).

As previously reported, a large WCA (θ > 90°) represents a hydrophobic surface, while a small WCA (θ < 90°) represents a hydrophilic surface [43]. To characterize their hydrophilicity/hydrophobicity, the WCAs of zein/millet gliadin nanofibers were tested. As illustrated in Figure 7, the WCAs of ZM-1/0 and ZM-0/1-1 are 120.88° and 111.35°, respectively, demonstrating that both the zein nanofibers and the millet gliadin nanofibers have hydrophobic surfaces, and that the zein nanofibers are more hydrophobic than the millet gliadin nanofibers. This is consistent with the findings regarding amino acid composition. Zein/millet gliadin composite nanofibers have WCAs of 120.97° and 117.84° when the zein/millet gliadin ratios are 1:1 and 1:2, indicating that the presence of millet gliadin does not significantly change the WCA of the zein matrix. Miri et al. [43] reported that incorporating AP into zein networks did not obviously alter the hydrophobic behavior of the nanofibers they used. However, compared with ZM-0/1-1, the WCA of ZM-0/1-2 is greatly increased, possibly because of its larger fiber diameter, which increases the surface roughness and thickness of ZM-0/1-2, thereby improving its surface hydrophobicity. These results suggest that the fiber diameter of a nanofiber contributes to its surface hydrophobicity. Yao, Chang, Ahmad, and Li [44] also reported that size distribution, as well as surface morphology, are essential determinants of the surface wettability of nanofibers.

#### 3.3.4. Water Stability of Nanofibers

**Figure 8:**
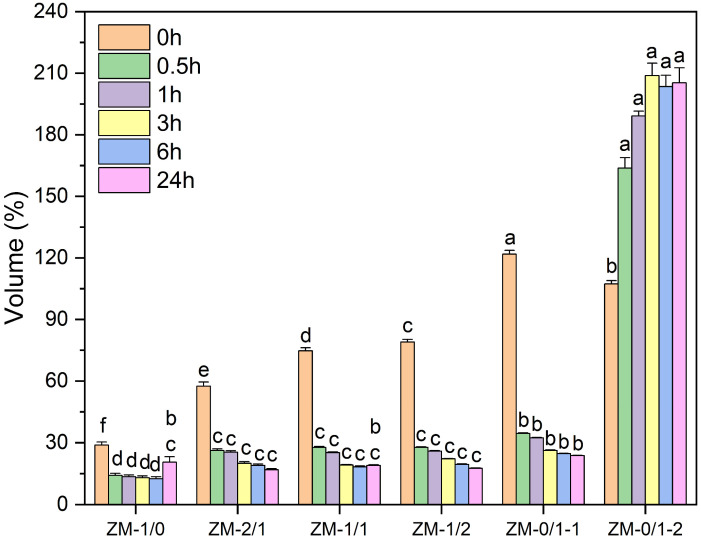
The volume changes of zein/millet gliadin nanofibers after immersion in water for 24 h. The various symbols on top of the columns suggest the significant differences (*p* < 0.05).

The water stability of electrospun fibers in aqueous media is important for their application. Therefore, zein/millet gliadin nanofibers were immersed in deionized water for 24 h to evaluate their water resistance, and the macroscopic images and volume changes of the nanofiber samples were investigated. As shown in Figure S2 and Figure 8, nanofibers with various zein/millet gliadin ratios exhibited distinct swelling behaviors. When initially in contact with water, ZM-1/0 underwent shrinking, and the volume became 59.37% of its initial value. Additionally, the volume of ZM-1/0 decreased with the increase in immersion time, showing a value of 20.60% after 24 h of water immersion. According to previous reports, this shrinkage probably results from the aggregating tendency of nanoscale hydrophobic domains [6]. In comparison, a remarkable reduction in the shrinkage of zein/millet gliadin composite nanofibers was observed when they were exposed to water; the volumes of ZM-2/1, ZM-1/1, and ZM-1/2 were measured at 57.46%, 74.72%, and 79.03%, respectively. Similarly, that volume gradually decreased with extended immersion time, reaching values of 16.91%, 19.03%, and 17.56%, respectively, after 24 h of water incubation. These results indicate that as the proportion of millet gliadin increases, the degree of shrinkage upon exposure to water of zein nanofibers decreases; however, when exposed for a long time, the improvement in water stability is no longer significant. However, ZM-0/1-1 and ZM-0/1-2 swelled upon initial contact with water, and the volumes became 121.86% and 107.31%, respectively. After water immersion for 24 h, the volume of ZM-0/1-1 decreased to 23.75%. Different from ZM-0/1-1, ZM-0/1-2 obviously increased in volume with the extension of immersion time, increasing to 205.29% after 24 h of water incubation. For millet gliadin nanofibers, the various swelling behaviors of ZM-0/1-1 and ZM-0/1-2 in water may be due to their differences in fiber diameter. Nanofibers with a smaller fiber diameter have a denser fiber structure, making them more prone to aggregation due to hydrophobic interactions after exposure to water.

**Figure 9:**
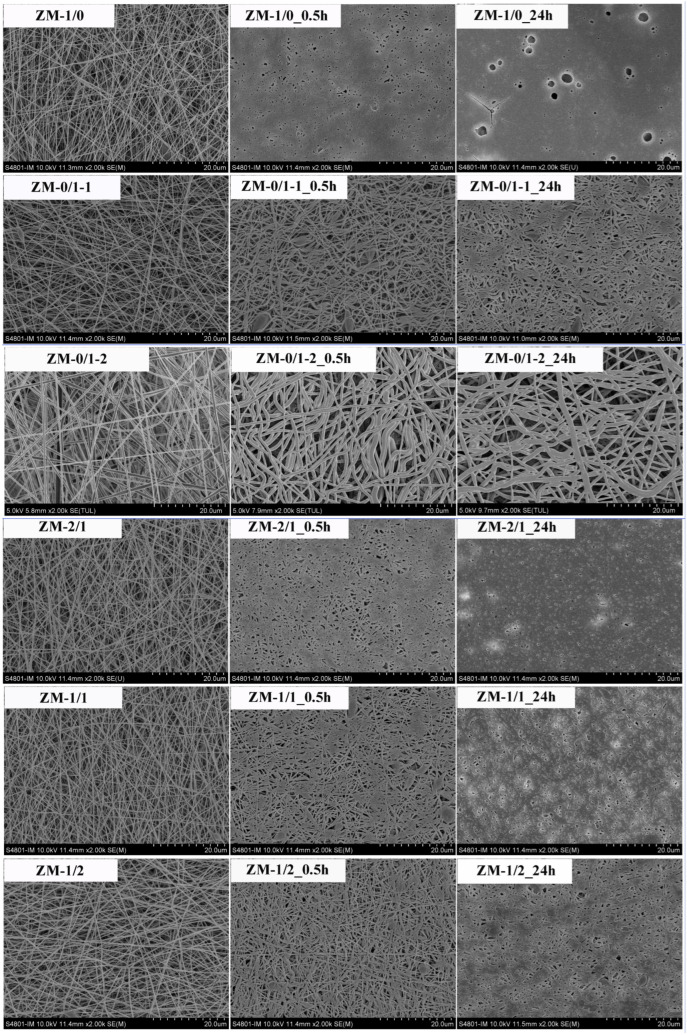
Scanning electron microscopy images of zein/millet gliadin nanofibers after increasing water immersion times up to 24 h.

To evaluate the swelling behavior and physical degradation of zein/millet gliadin nanofibers in aqueous media, the fiber morphologies of nanofibers immersed in water for different time periods were measured. As described in Figure 9, after immersion in water for 0.5 h, ZM-1/0 almost lost its original fiber structure, and the fibers aggregated to form a film-like layer with some pores formed by partially coalesced fibers. After 24 h of incubation, holes appeared on the surface of the membrane because of physical degradation. These results confirm the poor water stability of zein nanofibers. Lu et al. [32] also reported a similar result, observing that zein nanofibers were not water stable because of their high hydrophobicity. After immersion, ZM-0/1-1 still maintained a fibrous, porous structure, but the fibers were flattened and deformed. Nevertheless, after exposure to water, ZM-0/1-2 swelled because of water absorption, with slightly larger fiber diameters. These findings demonstrate that millet gliadin nanofibers are water stable, and that their water stability is closely related to their fiber diameters. It was observed that the water stability of zein nanofibers was improved by incorporating millet gliadin into the zein network. After immersion in water for 3 h, ZM-2/1 completely lost its fiber structure; for ZM-1/1, that time is 24 h. However, ZM-1/2 still maintained most of its fiber structure after 24 h of water incubation. In the work of Selling, Woods, Sessa, and Biswas [45], after wetting with water, zein nanofibers with glutaraldehyde (8%) cross-linking and heating (140 °C for 10 min) displayed a certain degree of water resistance.

#### 3.3.5. Thermogravimetric Analysis (TGA)

**Figure 10:**
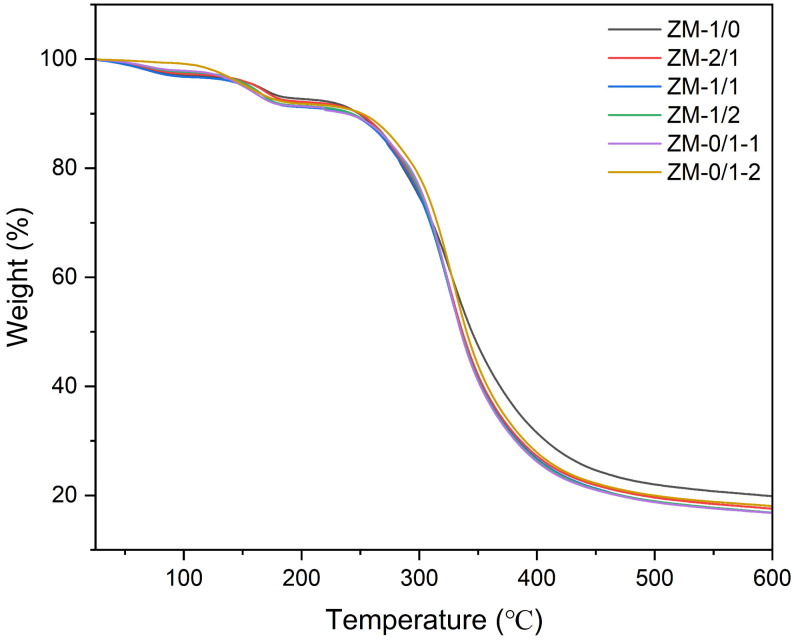
TGA curves of zein/millet gliadin nanofibers.

The thermal behavior of zein/millet gliadin nanofibers was investigated by TGA; the thermogravimetric results are presented in Table 5. As illustrated in Figure 10, the thermal degradation mainly consists of three steps. The initial 2–3% weight loss occurred in the range of 25 to 100 °C because of water evaporation. The 4–8% weight loss of the second stage happened in the range of 100–225 °C, which is related to the thermal degradation of low-molecular weight peptides. The major weight loss (72–82%), which is probably attributed to the thermal degradation and denaturation of zein and millet gliadin, occurred between 225 and 600 °C. The residual masses of ZM-1/0 and ZM-0/1-1 were 19.88% and 16.79%, respectively, suggesting that the thermal stability of zein nanofibers is higher than that of millet gliadin nanofibers. Ullah et al. [18] also reported a similar result, stating that the residue content of zein nanofibers at 500 °C was 23.31%. For zein/millet gliadin composite nanofibers, the residual mass at 600 °C decreased as the proportion of millet gliadin increased. Compared with ZM-0/1-1, a higher amount of residue was observed at 600 °C for ZM-0/1-2 (18.04%); this indicates a better thermal property, which is probably related to the increased entanglement in ZM-0/1-2 caused by its higher millet gliadin concentration. In the study by Wang et al. [46], it was observed that the presence of zein improved the thermal stability of konjac glucomannan nanofibers.

#### 3.3.6. Structures of Zein/Millet Gliadin Nanofibers

FTIR spectroscopy was carried out to study the chemical structure of zein/millet gliadin nanofibers. The peak located at around 1654 cm^−1^ was associated with the C=O stretching vibration, C–N stretching vibration, and N–H bending vibration of the amide I band [27,47]. As shown in Figure S3, the spectra of ZM-1/0 and ZM-0/1-1 show a peak at 1653 cm^−1^, while in the spectra of ZM-2/1, ZM-1/1, and ZM-1/2, a blue shift of that peak (1654 cm^−1^) was observed. Moreover, the peak intensity increased with the increasing proportion of millet gliadin. The increase in peak intensity shows evidence of the interaction between zein and millet gliadin [48]. Furthermore, the secondary structures of the zein/millet gliadin nanofibers were measured by deconvolution of the amide I band (1600–1700 cm^−1^). The correlations between peaks and sub-structures were as follows: α-helix at 1659–1648 cm^−1^, β-sheet at 1700–1680 cm^−1^ and 1640–1610 cm^−1^, β-turn at 1679–1660 cm^−1^, and random coil at 1648–1640 cm^−1^ [41]. As presented in Table 6, increasing the proportion of incorporated millet gliadin in the composite nanofibers resulted in an increase in the α-helix content and a decrease in the β-sheet content, which may be due to the increase in entanglement of the zein and millet gliadin molecules. It is considered that nanofibers with a high α-helix content generally possess a dense structure, which enables zein/millet gliadin nanofibers to exhibit high mechanical strength.

### 3.4. Composition Schematic Representation of Nanofibers

**Figure 11:**
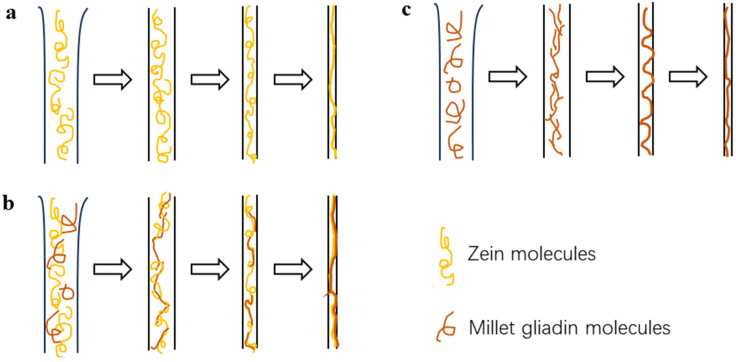
Schematic representation of electrospun zein fiber (**a**), zein/millet gliadin composite fiber (**b**), and millet gliadin fiber (**c**) formation.

The differences in mechanical properties between zein nanofibers and zein/millet gliadin composite nanofibers are probably due to the distinct chain entanglement behaviors of zein molecules and millet gliadin molecules in their nanofibers. A schematic representation of zein/millet gliadin nanofibers in acetic acid is presented in Figure 11. Compared to zein, millet gliadin molecules have a more unfolded structure in acetic acid due to their significantly smaller molecular weight. This sufficient unfolding is considered to allow more chain entanglement and continuous fiber formation [49]. During electrospinning, zein molecules and millet gliadin molecules are continuously stretched under electrostatic force. Since zein has shown characteristics similar to those of millet gliadin, their excellent compatibility allows enough chain entanglement of zein molecules with millet gliadin molecules. As the electrospinning process progresses, continuous entanglement, fusion, and stretching occur in zein and millet gliadin molecules, leading to the formation of zein/millet gliadin nanofibers with good mechanical properties. Therefore, the excellent compatibility, sufficient interaction (hydrogen bonding), and chain entanglement between zein and millet gliadin molecules led to the much higher mechanical strength of ZM-1/2 than that of ZM-0/1 and ZM-1/0.

## 4. Conclusions

In this study, zein nanofibers with significantly enhanced mechanical properties were successfully developed through the incorporation of millet gliadin. Because of the higher methionine and cysteine content, as well as the lower leucine content, millet gliadin had more intramolecular disulfide bonds, weaker hydrophobic interactions, and fewer intermolecular hydrogen bonds as compared with zein. Compared with zein, millet gliadin had a significantly lower molecular weight; furthermore, the more extended and elongated structure of acetic acid allowed the millet gliadin molecules to form stronger intermolecular interactions. For zein/millet gliadin composite nanofibers, ZM-1/2 showed the highest tensile strength of 49.32 MPa, which was 4.45 and 2.08 times stronger than that of pure zein and millet gliadin nanofibers, respectively. This increased mechanical strength is considered to result from the sufficient chain entanglement and compact structure formed for zein upon interaction with millet gliadin. The addition of millet gliadin to the zein network increased the water resistance of the zein nanofibers to some extent. These findings are expected to increase the application of zein nanofibers.

## Figures and Tables

**Table 1:** Formulations of electrospun zein/millet gliadin nanofibers.

Samples	Zein (g)	Millet Gliadin (g)	Eugenol (g)	Acetic Acid (mL)
ZM-1/0	30	0	0	100
ZM-2/1	20	8.33	0	100
ZM-1/1	15	12.50	0	100
ZM-1/2	10	16.67	0	100
ZM-0/1-1	0	25	0	100
ZM-0/1-2	0	30	0	100

**Table 2:** Amino acid compositions (g/100 g protein) and molecular weight profiles of zein and millet gliadin.

	Zein	Millet Gliadin
Amino acids (g/100 g protein)		
Glycine (Gly)	1.12	0.90
Alanine (Ala)	9.12	9.41
Proline (Pro)	6.51	5.28
Valine (Val)	1.63	2.17
Methionine (Met)	0.53	1.52
Isoleucine (Ile)	1.15	1.72
Leucine (Leu)	13.04	10.29
Phenylalanine (Phe)	4.62	3.53
Cysteine (Cys)	0.15	0.53
Tyrosine (Tyr)	3.38	2.13
Histidine (His)	0.78	0.86
Arginine (Arg)	0.92	0.87
Threonine (Thr)	1.10	1.64
Aspartic (Asp)	4.93	5.57
Glutamic acid (Glu)	19.95	18.22
Serine (Ser)	5.04	4.65
Lysine (Lys)	0.08	0.05
Hydrophobic amino acids	36.59	33.92
Hydrophilic amino acids	9.66	8.95
Molecular weight		
M_n_	5681	3401
M_w_	13,330	7623

**Table 3:** Secondary structure compositions of zein and millet gliadin.

Samples	Secondary Structure Composition (%)			
	α-Helix	β-Sheet	β-Turn	Random Coil
Zein	25.40 ± 0.45 ^b^	32.63 ± 0.63 ^a^	18.56 ± 0.39 ^d^	23.40 ± 0.21 ^c^
Millet gliadin	25.24 ± 0.05 ^b^	33.51 ± 0.11 ^a^	19.24 ± 0.03 ^d^	22.01 ± 0.09 ^c^

Results in each row with different lowercase superscript indicate significant differences (*p* < 0.05).

**Table 4:** Fiber diameters, fiber diameter distributions, and spans of zein/millet gliadin composite nanofibers.

Samples	Fiber Diameter (nm)	Fiber Diameter Distribution	Span
ZM-1/0	208.22 ± 47.04 ^a^	0.05	0.64
ZM-2/1	265.99 ± 54.87 ^a^	0.04	0.56
ZM-1/1	281.33 ± 49.78 ^a^	0.03	0.49
ZM-1/2	298.72 ± 55.39 ^a^	0.03	0.49
ZM-0/1-1	260.99 ± 49.75 ^a^	0.04	0.51
ZM-0/1-2	634.79 ± 152.73 ^b^	0.06	0.73

Results in each row with different lowercase superscript indicate significant differences (*p* < 0.05).

**Table 5:** Weight losses and residues of zein/millet gliadin nanofibers.

Samples	Weight Loss at 25–100 °C (%)	Weight Loss at 100–225 °C (%)	Weight Loss at 225–600 °C (%)	Residue at 600 °C (%)
ZM-1/0	2.85 ± 0.11 ^b^	4.45 ± 0.08 ^e^	72.59 ± 0.21 ^c^	19.88 ± 0.17 ^a^
ZM-2/1	2.68 ± 0.06 ^b^	5.05 ± 0.10 ^d^	74.41 ± 0.19 ^b^	17.58 ± 0.16 ^c^
ZM-1/1	3.25 ± 0.08 ^a^	5.37 ± 0.14 ^c^	74.15 ± 0.20 ^b^	16.83 ± 0.18 ^d^
ZM-1/2	2.36 ± 0.12 ^c^	6.17 ± 0.19 ^b^	74.41 ± 0.24 ^b^	16.84 ± 0.19 ^d^
ZM-0/1-1	2.13 ± 0.10 ^d^	6.40 ± 0.17 ^b^	74.46 ± 0.18 ^b^	16.79 ± 0.21 ^d^
ZM-0/1-2	0.87 ± 0.13 ^e^	7.70 ± 0.15 ^a^	81.96 ± 0.26 ^a^	18.04 ± 0.19 ^b^

Results in each row with different lowercase superscript indicate significant differences (*p* < 0.05).

**Table 6:** Secondary structure compositions of zein/millet gliadin nanofibers.

Samples	Secondary Structures (%)		
	β-Sheet	α-Helix	β-Turn
ZM-1/0	42.87 ± 0.15 ^c^	33.55 ± 0.35 ^d^	23.58 ± 0.45 ^a^
ZM-2/1	43.84 ± 0.15 ^b^	33.87 ± 0.41 ^d^	22.29 ± 0.46 ^b^
ZM-1/1	41.32 ± 0.29 ^d^	34.93 ± 0.52 ^c^	21.49 ± 0.32 ^c^
ZM-1/2	41.29 ± 0.47 ^d^	35.37 ± 0.46 ^c^	21.58 ± 0.23 ^c^
ZM-0/1-1	48.48 ± 0.39 ^a^	37.14 ± 0.40 ^b^	11.15 ± 0.37 ^e^
ZM-0/1-2	40.07 ± 0.38 ^e^	39.83 ± 0.35 ^a^	17.38 ± 0.41 ^d^

Results in each row with different lowercase superscript indicate significant differences (*p* < 0.05).

## Data Availability

The original contributions presented in the study are included in the article/Supplementary Materials, further inquiries can be directed to the corresponding authors.

## Supplementary Materials

The following supporting information can be downloaded at: https://www.mdpi.com/article/10.3390/foods13182900/s1, Figure S1: FTIR spectra of zein and millet gliadin; Figure S2: Images of zein/millet gliadin nanofibers after immersion in PBS for 24 h; Figure S3: Fourier transform infrared spectra of zein/millet gliadin composite nanofibers; Figure S4: Scanning electron microscopy images of ZM-1/0 after increasing water immersion times up to 24 h; Figure S5: Scanning electron microscopy images of ZM-0/1-1 after increasing water immersion times up to 24 h; Figure S6: Scanning electron microscopy images of ZM-0/1-2 after increasing water immersion times up to 24 h; Figure S7: Scanning electron microscopy images of ZM-2/1 after increasing water immersion times up to 24 h; Figure S8: Scanning electron microscopy images of ZM-1/1 after increasing water immersion times up to 24 h; Figure S9: Scanning electron microscopy images of ZM-1/2 after increasing water immersion times up to 24 h.
