# Supplementary material for: Enhancement of Mechanical Properties of Zein-Based Nanofibers by Incorporation of Millet Gliadin
Source: Foods. 2024 Sep 13;13(18):2900. doi: 10.3390/foods13182900 (PMC11431402; doi:10.3390/foods13182900)
Supplement: Supplementary file 1 [file foods-13-02900-s001.zip › foods-3175500-supplementary.pdf]

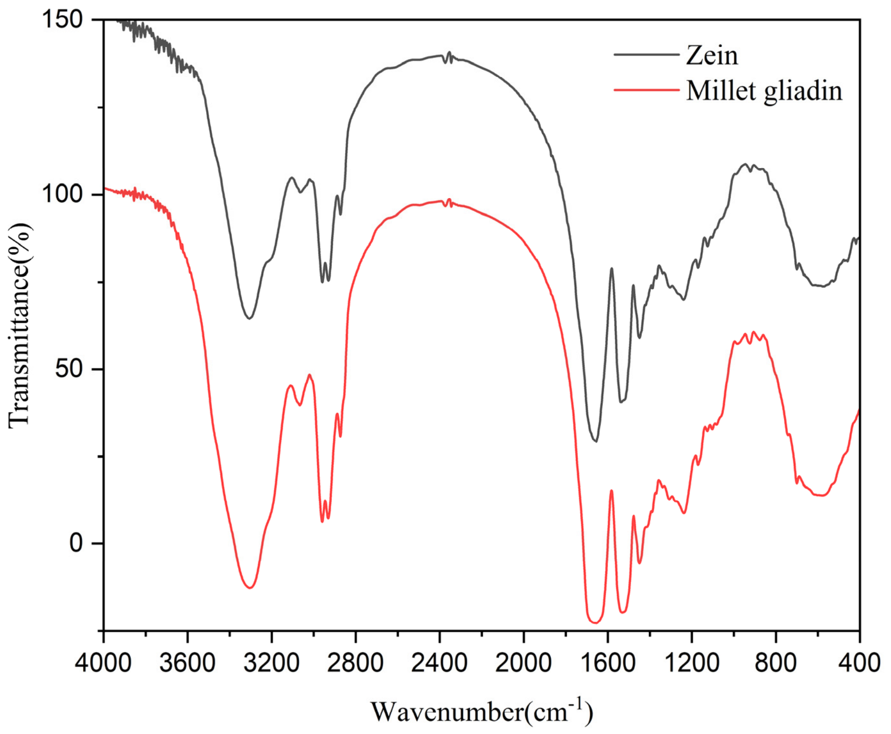

Figure S1. FTIR spectra of zein and millet gliadin.

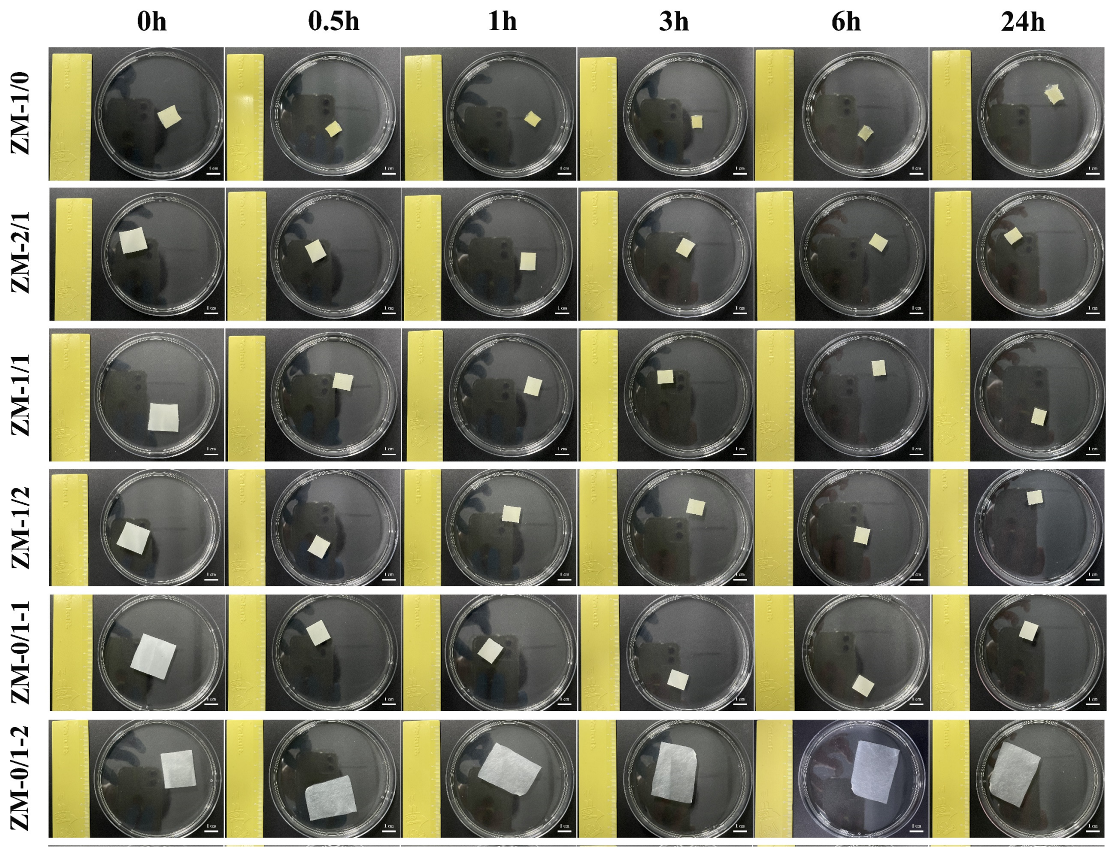

Figure S2. Images of zein/millet gliadin nanofibers after immersion in PBS for 24 h.

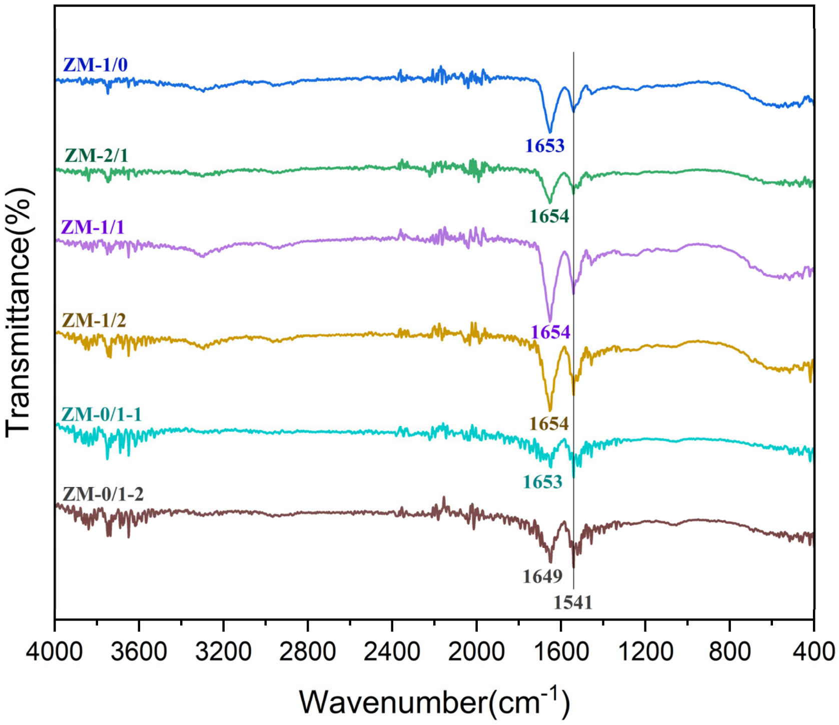

Figure S3. Fourier transform infrared spectra of zein/millet gliadin composite nanofibers.

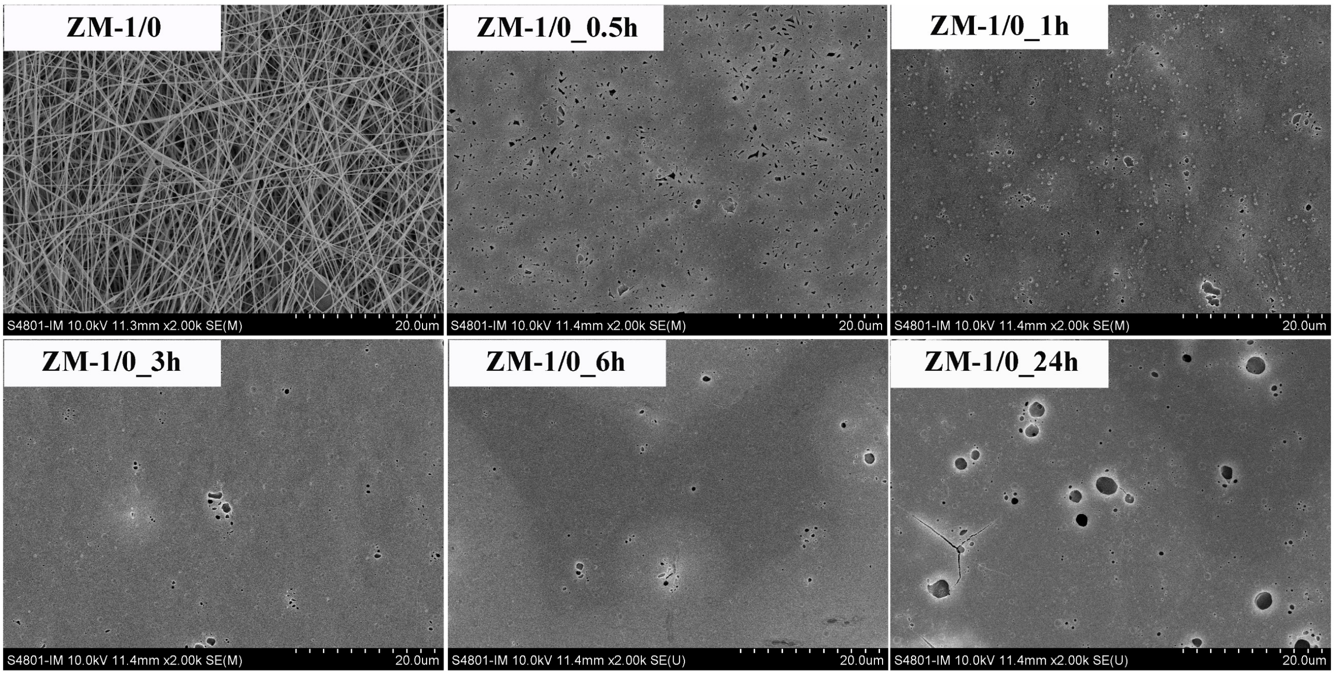

Figure S4. Scanning electron microscopy images of ZM-1/0 after increasing water immersion times up to 24 h.

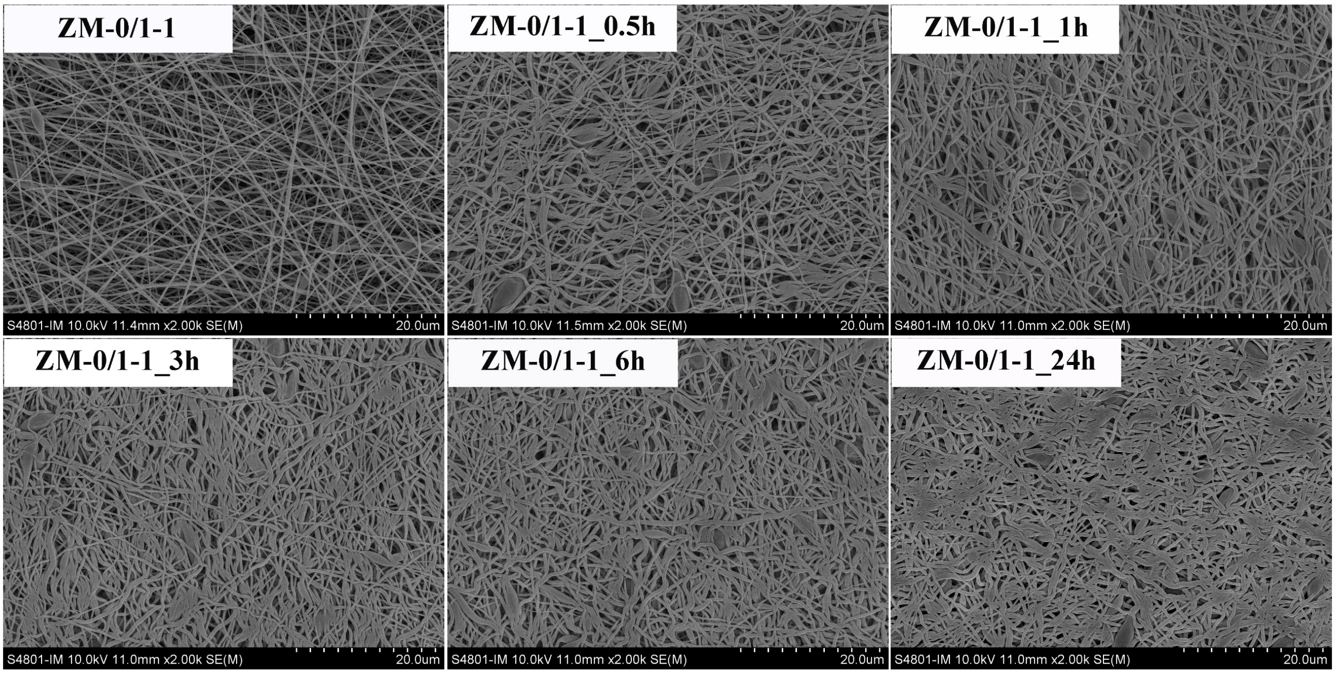

Figure S5. Scanning electron microscopy images of ZM-0/1-1 after increasing water immersion times up to 24 h.

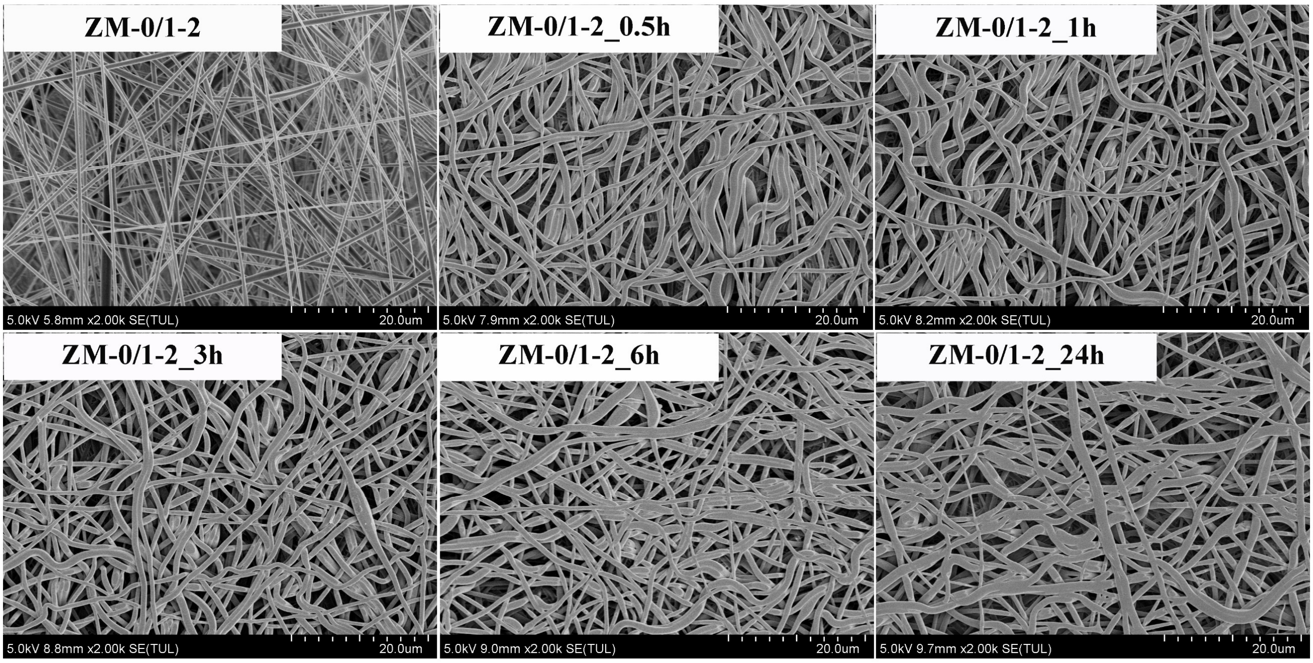

Figure S6. Scanning electron microscopy images of ZM-0/1-2 after increasing water immersion times up to 24 h.

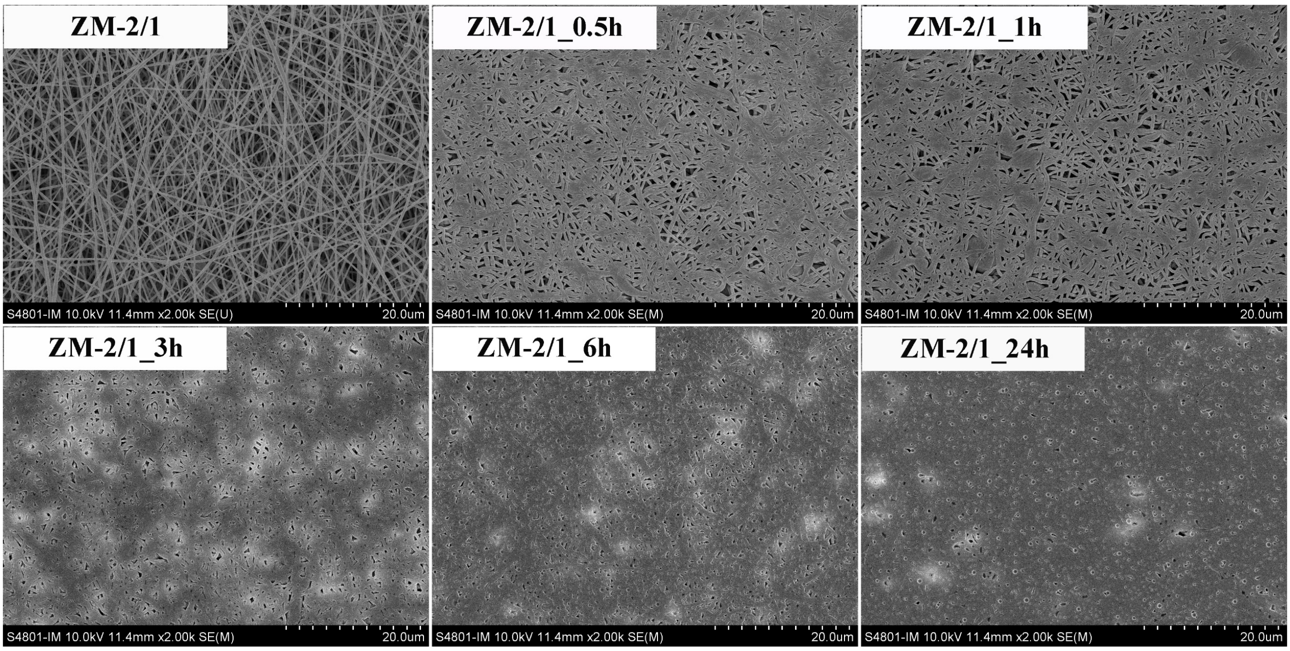

Figure S7. Scanning electron microscopy images of ZM-2/1 after increasing water immersion times up to 24 h.

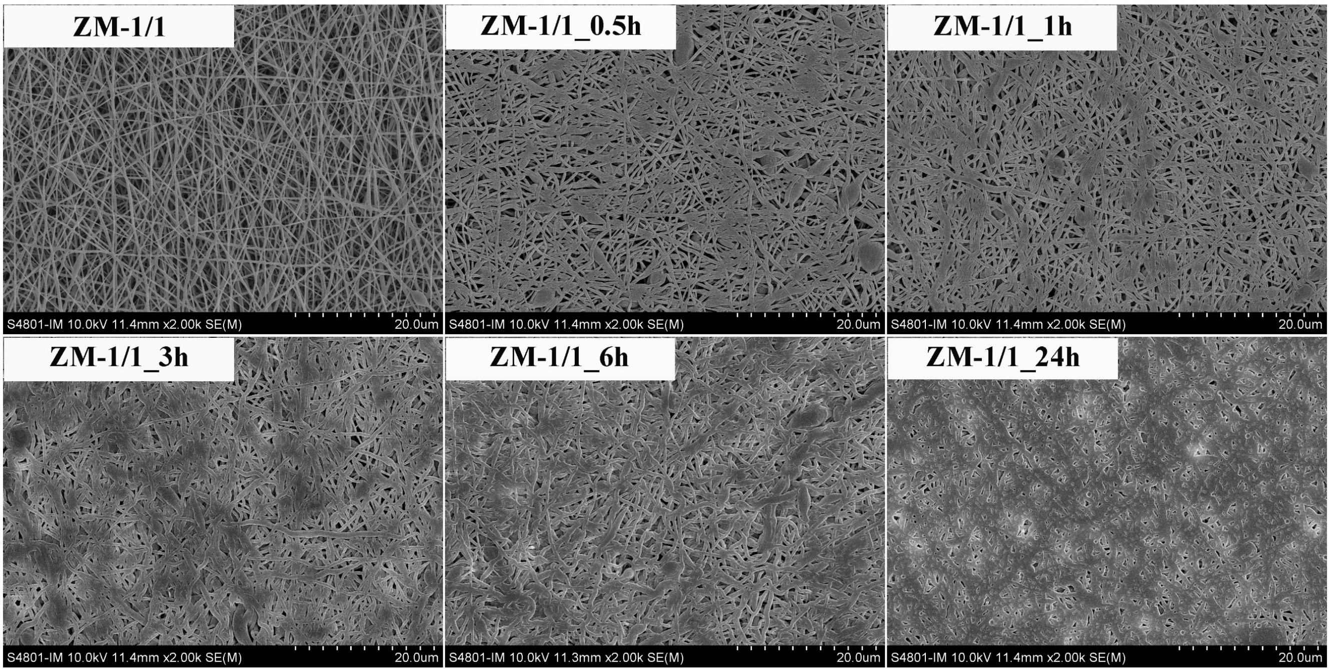

Figure S8. Scanning electron microscopy images of ZM-1/1 after increasing water immersion times up to 24 h.

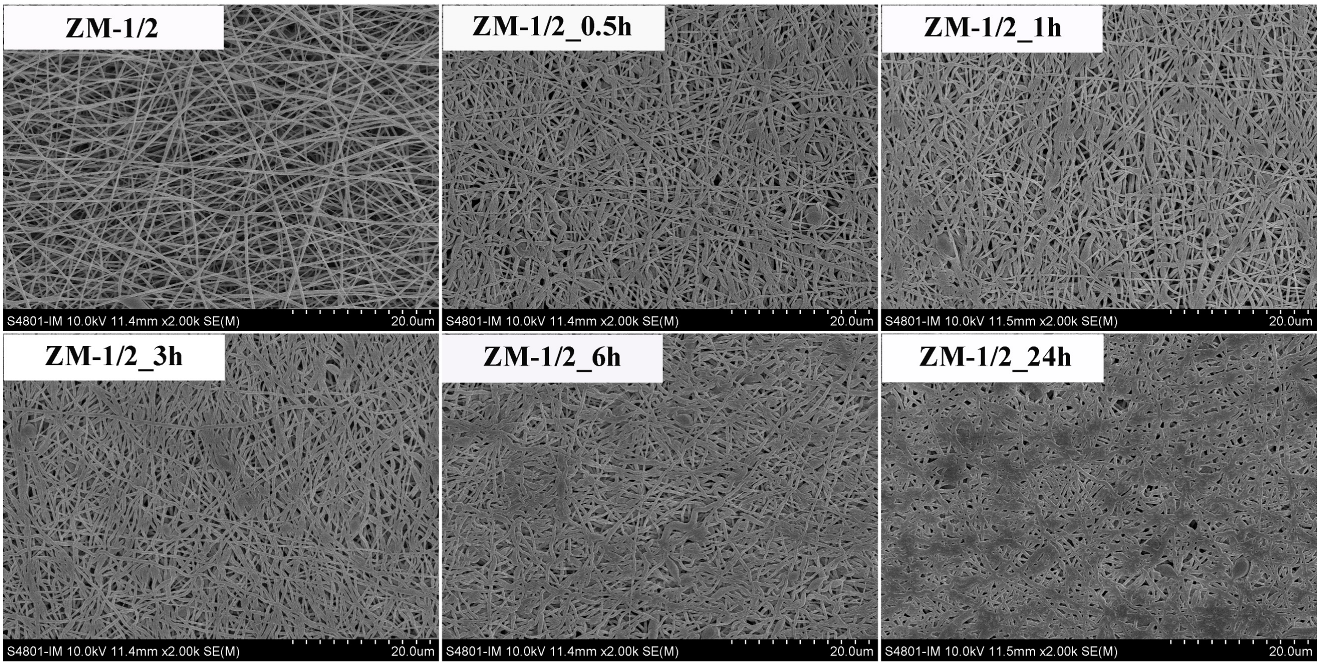

Figure S9. Scanning electron microscopy images of ZM-1/2 after increasing water immersion times up to 24 h.
